# 

**Published:** 1910-02-12

**Authors:** 


					February 12, 1910. THE HOSPITAL. 581
Gifts and Bequests.
The Hospital for Sick Children, Great Ormond Street,
has received 100 guineas from the Royal Warrant Holders'
Association.
The net estate of Mr. George Salting, the art collector,
has been sworn at ?1,287,906 6s. lOd. He left ?10,000 to
the London hospitals and ?2,000 to the Prince Alfred Hos-
pital, Sydney, N.S.W.
Mrs. Elizabeth Bannister, after various bequests,
left tha residue of her estate in equal shares to the
Chelsea Hospital for Women, the Hospital for Consump-
tion at Brompton, and six other charities.
The Lying-in Hospital of New York has received from
Mrs. Helen Hartley Jenkins $25,000 to enable the hospital
to make a special study of puerperal fever. The fund is to
he known as the Emma Hartley Endowment.
By the will of the late Anna Eliza Porter, of Philadelphia
the sum of $10,000 is bequeathed to the Methodist Epis-
Copal Hospital for a free room to be known as the Porter
Memorial, with $200 aditional for the equipment of the
room.
Mrs Lydia Husband, of Grange-over-Sands, after mak-
lng certain bequests, has left the residue of her property to
her sister for life, with remainder equally between the Sal-
ford Royal Hospital and Dispensary and the Children's
Hospital, Pendlebury.
Mr. James Smith, cotton broker, has left ?10,COO in
trust for religious and charitable institutions in or around
Liverpool, ?1,000 to the David Lewes Northern Hospital,
Liverpool; ?1,000 each to the Wallasey Cottage Hospital,
Victoria Central Hospital, Liscara.
Mrs. Mary Williamson has left ?1,000 to the District
Infirmary, Achton-under-Lyne; ?500 to the Northern
Counties Hospital for Incurables, Heaton Mersey; ?200
each to the Manchester Royal Eye Hospital and the
General Hospital and Dispensary for Sick Children,
^ndlebuvy.
Mr. Samuel William Eden has left ?250 each to the
Nottingham General Hospital, the Nottingham Samaritan
hospital for Women, the Nottingham and Midland Eye
Infirmary, and the Nottingham Children's Hospital, and
?50 to the Nottingham Association for the Prevention of
Consumption.
Miss Mathilde Dresden, of Cavendish Square, W., has
bequeathed a sum equal to two years' subscriptions to each
charitable institution to which she had subscribed for two
.Years before her death; and ?500 to the Middlesex Hoe-
pital, ?200 being for the Medical School and ?300 for
8eneral purposes.
Among the latest contributions for King Edward's Hos-
pital Fund for London are the following annual subscrip-
tions : Mr. Walter Morrison, ?5,000; Mr. William Wal-
dorf Astor, ?1,000; Messrs. C. J. Hambro and Son, ?250;
Prudential Assurance Company, Limited, ?250; Messrs.
^ernher, Beit and Co., ?250; Mrs. Walter H. Burns,
?200.
Mr. George W. Long, of Swanage, should a legatee to
^hom he bequeathed ?500 on trust for life leave no issue,
has left ?300 equally between the Cancer Hospital, Bromp-
t'On, the London S.P.C. among the Jews, and the Royal
Hospital for Incurables, Putney; and his shares in two
Sae companies to the Yeatman Hospital, Sherborne, for the
Samaritan Fund. Subject to his wife's life interest, he left
?250 4 per cent, stock of the L.N.W.R. Co. to the Yeatman
Hospital.
Mr. Henry Percival Stebbing has left ?300 to the Home
and Infirmary for Sick Children, Lower Sydenham, ?100
to the Forest Hill Provident Dispensary, ?200 to the Metro-
politan Convalescent Institution for the Bexhill Branch,
and ?100 to the same institution for the Children's Branch
at Broadstairs.
Tiie Royal National Orthopaedic Hospital has received,
among recent contributions, ?5,000 from King Edward's
Hospital Fund (final instalment of ?15,000), ?100 each
from Lord Iveagh, the City Corporation, and Mr. C. H.
Oliverson; ?26 5s. from the Saddlers' Company; ?25 from
Sir Julius Wernher; and ?21 from the Skinners' Company.
A donation of ?1,000 to the Manchester Royal Infirmary
has been announced from Mr. George Spiegelberg, of
Bowdon, for the purpose of endowing a bed in the male
cection for cancer patients, to be known as the " Edward
and Antonia Spiegelberg bed," available for any male
patient suffering from cancer, irrespective of nationality
or creed.
The Leicester Infirmary and Children's Hospital has
received intimation of the following legacies : from Mr.
Alexander Ross of Leicester ?100, and another ?100 to
the endowment fund of the Children's Hospital. From the
estate of Miss M. A. Coleman a moiety estimated at ?600.
From the estate of Mr. Charles Turner of Leicester ?1,400:
on account of a two-thirds share.
Mr. Henry Minsox has left a sum estimated at about
?400 to each of the following institutions : the Royal Hos-
pital for Incurables, Putney Heath; the London Fevei-
Hospital, Liverpool Road, Islington, N.; the Metropolitan
Hospital, Kingsland Road, N.; the London Hospital, the
Great Northern Central Hospital, Holloway Road, N.; and
the Royal Sea Bathing Hospital, Margate.
The Treasurer of St. Bartholomew's Hospital acknow-
ledges the following further contributions to the special
appeal issued by the Lord Mayor, namely :?Union Assur-
ance Society (Ltd.), ?500; Peninsular and Oriental Steam
Navigation Company, ?52 10s.; Mr. Charles T. D. Crews,
?52 10s. ; Anonymous, ?52 10s. ; Anonymous, ?52 10s. ;
Anonymous, ?25; Miss Ella Mocatta, ?10 10s.; Mrs.
Walch, ?10 10s.
The value of the bequests for public uses under wills
proved last month exceeds ?1,000,000. Under 80 of the
wills of 1909 there were public bequests amounting in all
to about ?3,500,000, making a total for the four years of
nearly ?16,500,000 under the wills of 310 testators whose
estates were valued at ?58,500,000. The total amount of
the public bequests during the four years would probably
be about ?18,500,000, with a yearly average of more than
?4,500,000.
The Cutlers' Company has granted ?10 10s. to each of
the following : Royal National Hospital for Consumption
and Diseases of the Chest, Seaside Convalescent Hos-
pital, Seaford, Royal Sea Bathing Hospital, Margate,
Royal Dental Hospital, Central London Ophthalmic
Hospital, Queen's Hospital for Children, Royal "National
Orthopaedic Hospital, National Hospital for Para-
lysed and Epileptic, Charing Crocs Hospital, Victoria
Hospital for Sick Children, London Hospital, Middlesex
Hospital, Royal Hospital for Incurables, Putney, London
Throat Hospital, St. Mary's Hospital, Ilford, East-end
Mothers' Lying-in Home, Brentwood Cottage Hospital,
Friedenheim Hospital, Children's Hcspital for Hip Disease,
Sevenoaks, and Evelina Hospital.
Appointments.
Dr. E. Willis has been appointed medical officer to
the Cranleigh district.
Dr. T. Aubrey has been appointed medical officer under
the Warmley District Council.
Dr. Hugh Macintyre has been appointed medical officer
of health to the parish of Kilarrow.
Dr. Arthur Latham has been elected a physician to the
Mount Vernon Hospital for Consumption and Diseaees of
the Chest, Hampstead and Northwood.
Dr. E. B. Leech has been appointed medical registrar,
and Dr. F. G. Wrigley reappointed at the Manchester
Royal Infirmary as house surgeon.
The West London Hospital, Hammersmith Road, has ap-
pointed Mr. Aslett Baldwin, F.R.C.S. (England), to be
surgeon ; Mr. Raymond Broadley Etherington-Smith, M.A.,
M.B., B.C. (Cantab), F.R.C.S. (England), to be assistant
surgeon,; and Mr. Oswald Lacy Addison, M.B., B.S.,
(London), F.R.C.S. (England), to be honorary surgical
registrar.
THE QUESTION BOX.
SPECIAL NOTICE.?Questions of interest affecting" the honorary
medical staff and hospital administration in all departments
are invited. When any point arises we hope our readers will
formulate it in a question and so co-operate to make this
column of real value and interest. In this way the experience
of the best-managed hospitals should be made helpful to the
whole hospital world. We shall welcome the contribution of
both questions and answers.
N-B.?The publication of an answer in this column does not
necessarily preclude the publication of subsequent answers to
the same question, for a question may involve points which
require to be threshed out and fully discussed. Answers, if
published, will be paid for at scale rates.
WANTED, A SYSTEM FOR FILING.
Question : What is the best system for filing
records, documents, and letters suitable for a large
hospital?
A Liverpool View.
By Mr. Francis H. Moore, General Superintendent and
Secretary, Liverpool Royal Infirmary.
I have had considerable and varied experience of "office
routine," irrespective of my hospital life, and a simple
plan of " filing " documents so that those which may have
to bo consulted during the absence of the chief can be
readily found, and one not likely to become cumbersome
by accumulation has not failed to engage my attention.
I do not advocate the systems which nececeitate the
punching of holes and the tiling of every letter received.
My practice is to divide letters and documents received
into two divisions?these likely to be subsequently re-
ferred to, and these which aro not so. The first are com-
paratively few, and are indexed; the others, with every
memoranda, are placed in a receptacle, the contents of
which, when full, are tied together, and labelled with the
inclusive dates, and are put aside for a time before they
are sent to the lumber room. There are other papers
which can be grouped and " pigeon-holed " for the time
during which the subjects to which they relate are likely
to be under consideration. Afterwards these can be tied
together, and eventually sent to the lumber room; but a
considerable period should be allowed to elapse before any
are destroyed.
Of course "historical documents" should never te
destroyed or placed with lumber, but kept under lock and
key in a fireproof safe.
THE USES.OF AN ADDRESSOGRAPH.
Question 1: Is it an advantage for a hospital to
possess an addressograph ? if so, describe tho
advantages and give the particulars of cost.
Mr. John Wortley Axe, late Professor in the Royal
College of Veterinary Surgeons, London, has left ?2,000
to the Victoria Veterinary Benevolent Institution upon
trust to accumulate at compound interest till it totals
?3,000, and then with ?700 to purchase land and two cot-
tages and to apply the income from the remaining ?2,300
in allowances to inmates who are to be either members or
past members of the R.C.V.S., who from age, accident,
or diseace are unable to follow their profession, or to their
aged or infirm widows. He left also ?400 to the R.C.V.S.,
Camden Town, N.W., for full-length portraits of Pro-
fessors Spooner, Summers, Varnell, and Brown, to be hung
in the council chamber in recognition of their great services
to veterinary science.
586 THE H0SP1TA L. February 12, 1910.
THE
GRADUATES' MEDICAL SCHOOLS.
DIARY FOR THE WEEK, FEBRUARY 14 to 19.
CENTRAL LONDON THROAT AND EAR
HOSPITAL, Gray's Inn Road, W.C.
At 3.45 p.m.?Feb. 15, Mr. Cliichele Nourse, External Ear
and Labyrinth.
4.30 p m. ? Feb. 17, Dr. Dan McKenzie, When to
Operate in Middle Ear Suppuration.
3.45 p.m.?Feb. 18, Mr. Cliichele Nourse, External Ear
and Labyrinth.
MEDICAL SOCIETY OF LONDON, Chandos Street,
Cavendish Square, W.
At 8 30 p.m.?Feb. 14, Dr. A. H. X. Lewes. A Case of
Pan-hysterectomy for Carcinoma of the Body of the
Uteruf, in which an Infiltrated Portion of the Bladder
was removed. Dr. Victor Bonney, Treatment of
Uterine Haemorrhage.
THE BROMPTON HOSPITAL FOR CONSUMP-
TION AND DISEASES OF THE CHEST,
Fulham Road, S.W.
At 4 p.m.?Feb. 16, Dr. Jex Blake, Fibroid Lung.
NORTH-EAST LONDON POST-GRADUATE COL-
LEGE, Prince of Wales's General Hospital,
Tottenham, N.
At 4 30 p.m.?Feb. 15, Dr. G. P. Chappel, Demonstration
of Medical Cases.
Feb. 17, Dr. Kenneth Kellie, The Feeding of Infants.
MEDICAL GRADUATES' COLLEGE AND
POLYCLINIC, 22 Chenies Street, W.C.
At 4 p.m.?Feb. 14, Dr. George Fernet, Clinique (Skin).
5.15 p.m.?Feb. 14, Dr. E. C. Bousfield, Recent De-
velopments in Serum Diagnosis.
4 p.m.?Feb. 15, Dr. Newton Pitt, Clinique (Medical).
5.15 p.m.?Feb. 15, Mr. A. II. Tubby, Lateral Curva-
ture of the Spine (continued).
4 p.m. Feb. 16, Mr. A. II. Tubby, Clinique (Surgical).
5.15 p.m.?Feb. 16, Dr. George Caip?nter, Heart Disease
in Children.
4 p.m.?Feb. 17, Sir Jonathan Hutchinson, Clinique
(Surgical).
5.15 p.m.?Feb. 17, Dr. Clive lliviere, Conditions which
Simulate Phthisis in Children.
4 p.m.?Feb. 18, Dr. W. H. Kelson, Clinique (Ear,
Nose, and Throat).
ST. JOHN'S HOSPITAL FOR DISEASES OF THE
SKIN, Leicester Square, W.C.
At 6 p.m.?Feb. 17, Chesterfield Lecture: Acne Vulgaris,
LONDON SCHOOL OF CLINICAL MEDICINE,
Seamen's Hospital, Greenwich, S.E.
At 2.15 p.m.?Feb. 14, Mr. William Turner, Burns.
2.15 p.m.?Feb. 15, Dr. Kussell Wells, Anaemia.
THE POST-GRADUATE COLLEGE, West London
Hospital, Hammersmith, W.
At 10 a.m.?Feb. 14 and Feb. 17, Demonstration by Sur-
gical Registrar.
10 a.m.?Feb. 18, Demonstration by Medical Registrar.
12 noon.?Feb. 17, Dr. .Bernstein, Pathological Demon-
stration.
1215 p.m.?Feb. 15, Dr. Pritchard, Practical Medicine-
12.15 p.m.?Feb. 16, Dr. Grainger Stewart, Practical
Medicine.
5 p.m.?Feb. 14, Dr. Eobinson, Gyniecology.
5 p.m.?Feb. 15, Mr. 1'ardoe, Clinical Lecture.
5 p.m.? Feb. 16, Dr. Beddard, Medicine.
5 p.m.?Feb. 17, Mr. Edwards, Rectal Surgery.
5 p.m.?Feb. 18, Dr. Abraham, Cases of Skin Disease.
ROYAL SOCIETY OF MEDICINE, 20 Hanover
Square, W.
At 8.30 p.m.?Feb. 15, Pathological Section:
Papers.?Dr. H. R. Dean : " A note on some of the factors
concerned in serum diagnosis of syphilis." Mr. E.
Groves : "A case of spontaneous fracture arising from a
condition of osteitis of obscure nature." Mr. S. H-
Shattock and Mr. L. S. Dudgeon: "Experiments in
grafting foetal cartilage."
At 5 p.m.?Feb. 17, Dermatological Section:
Clinical Cases.?Dr. J. H. Sequeira : Lupus disseminatus
faciei. Dr. Vincent Dickinson: A case for diagnosis.
Mr. Willmott Evans : Sclerodermia pigmentosa. And
other eases.
At 8.30 p.m.?Feb. 18. Electro=Therapeutical Section :
Papers.?Dr. Thurstan Holland : " Points in the diagnosis
of ureteral calculi." Dr. Howard Humphris : "The
static wave current and other forms of static electricity.'
Abdulla and Co., Ltd., cigarette specialists, of 9 Bond
Street, W., if sue a useful and attractively-got-up list of
members of the new House of Commons. The little list
is accurate, and contains much information which will be
of use for reference purposes.

				

